# Synthesis of Copper Telluride Thin Films by Electrodeposition and Their Electrical and Thermoelectric Properties

**DOI:** 10.3389/fchem.2022.799305

**Published:** 2022-01-21

**Authors:** Jungjoon Park, Jinmyeong Seo, Jae-Hong Lim, Bongyoung Yoo

**Affiliations:** ^1^ Department of Materials Science and Chemical Engineering, Hanyang University, Ansan, South Korea; ^2^ Department of Materials Science and Engineering, Gachon University, Seongnam, South Korea

**Keywords:** copper telluride, electrodeposition, thermoelectric, compositional control, carrier filtering effect

## Abstract

Intermetallic copper telluride thin films, which are important in a number of electronics fields, were electrodeposited using a potentiostatic method in low-pH aqueous electrolyte baths with various ion-source concentrations, and the electrical properties of the formed films were investigated after exfoliation from the substrate. The films were electrochemically analyzed by cyclic voltammetry, while surface and cross-sectional morphologies, compositional ratios, and electrical properties were analyzed by scanning electron microscopy, X-ray diffractometry, X-ray photoelectron spectroscopy, ultraviolet photoelectron spectroscopy, and Hall-effect experiments. The copper telluride thin films, which were synthesized at various potentials in each bath, exhibit different composition ratios and structures; consequently, they show a variety of electrical and thermoelectric properties, including different electrical conductivities, carrier concentrations, mobilities, and Seebeck coefficients. Among them, the thin film with a 1:1 Cu:Te ratio delivered the highest power factor due to carrier filtering at the interface between the two phases.

## Introduction

Copper telluride has attracted increasing attention in recent years for potential thermoelectrics, quantum dot, battery, and plasmonics applications because of its p-type semiconductor properties (Kriegel et al., 2013; Nethravathi et al., 2014; Han et al., 2014; Zhang et al., 2016). However, synthesizing intermetallic semiconductors with exact CuTe and Cu_2_Te stoichiometries is difficult, which is mainly due to copper vacancies; hence, copper telluride exists in a wide range of nonstoichiometric Cu_2-x_Te phases, including Cu_1.5_Te, Cu_7_Te_5_, and Cu_7_Te_4_. Hence, the characteristics of a material can potentially be controlled by adjusting the copper telluride composition and structure.

Various methods have been developed for the synthesis of copper telluride, including hydrothermal, solvothermal, ion-exchange, vacuum-deposition, and electrodeposition techniques (Zhang et al., 2006; Dhasade et al., 2012; Kriegel et al., 2013; Nethravathi et al., 2014; Bhuvaneswari et al., 2017). Hydrothermal synthesis involves the decomposition of reactants or chemical reactions between them at high temperature and high pressure in an aqueous solution, while solvothermal synthesis is similar but involves a non-aqueous solvent (Nethravathi et al., 2014). These two synthesis methods are advantageous because they lead to nanostructures with uniform crystalline phases that are not stable at their melting points; however, they require expensive autoclaves, are associated with safety issues during reaction, and observing reaction process is impossible as the reaction proceeds in a “black box.” Ion-exchange synthesis involves the exchange of ionic components (Kriegel et al., 2013) and is widely used to prepare nanostructured metal chalcogenides because their properties can be controlled through cation or anion exchange. However, it has some drawbacks, including long production times and large pH changes during reaction (Chen and Wang, 2017; Cho et al., 2019). While these methods are outstanding for the synthesis of nanostructured materials, they require the use of additional processes, such as sintering, for thermoelectric applications (Zhang et al., 2020).

Vacuum-deposition processes, which include sputtering, e-beam evaporation, and chemical vapor deposition, are used to deposit thin films at pressures below atmospheric pressure (Bhuvaneswari et al., 2017) and are advantageous because they deposit good-quality thin films. However, some processes require expensive equipment while others require long production times. In contrast, electrodeposition involves the electrochemical reduction of metal ions in solution and has benefits that include high throughput, the formation of highly pure deposited films, wide deposition areas, low operating temperatures, and relative cost effectiveness. Electrodeposition can be used to tailor the composition of the deposited material by adjusting its wide range of controllable parameters, such as the concentration of the ion source, application potential, applied current density, solution pH, additives, and the electrical wave form, which provide outstanding advantages. These electrodeposition characteristics can be used to control the composition and stoichiometry of copper telluride, which has numerous metastable states.

In this study, low-pH electrolytes were used to synthesize copper telluride thin films by electrodeposition, with the composition of the electrodeposited thin films controlled by adjusting the concentration of the ion source and the applied potential. The electrical properties of the electrodeposited intermetallic copper telluride thin films, including carrier concentration, conductivity, mobility, and thermoelectric properties, were investigated by mechanically transferring the films from the original substrate to epoxy resin.

## Materials and Methods

### Electrochemical Cell for Copper Telluride Electrodeposition

The electrolyte for copper telluride electrodeposition was prepared by dissolving Cu(NO_3_)_2_·3H_2_O (Daejung) and TeO_2_ (Sigma–Aldrich) in a mixture of deionized water and nitric acid. Copper nitrate was selected as the copper salt based on a previous study on Cu_2-x_Te-film electrodeposition using various copper metal salts with various anions, such as sulfate, chloride, and nitrate (Zhang et al., 2011). First, 60% nitric acid (20 ml) was added to deionized water (80 ml) to form a pH 1.0 solution because TeO_2_ has limited solubility in highly alkaline aqueous solutions, after which Cu(NO_3_)_2_ was dissolved in the solution to a concentration of 10, 7.5, or 5 mM. Finally, TeO_2_ was dissolved in the solution to a concentration of 5, 7.5, or 10 mM) as the Te-ion source. The total ion concentration (Cu + Te) was 15 mM in each case.

A three-electrode system consisting of a Ag/AgCl reference electrode (Fisher Scientific, 13-620-53), a Pt plate (20 mm × 130 mm) counter electrode, and a Si/SiO_2_/Ti/Au wafer working electrode was used to electrodeposit CuTe. Each wafer sample was cleaved to be 12 mm × 10 mm in size. Prior to electrodeposition, cyclic voltammetry (CV) was used to understand the electrochemical reaction of Cu and Te on the Au substrate surface. A VersaSTAT MC potentiostat/galvanostat (Princeton Applied Research) was used for all electrochemical syntheses and analyses. Electrodeposition was performed at room temperature.

### Characterizing the Electrodeposited Thin Films

The surface morphologies, film thicknesses, and compositions of the deposited thin films were investigated by scanning electron microscopy (SEM; TESCAN, MIRA3) augmented with energy-dispersive X-ray spectroscopy (EDS). The phase-formation behavior and crystal orientations of the films were investigated by conventional X-ray diffractometry (XRD; Rigaku D Max-2500) using Cu Kα radiation. The microstructure was elucidated by transmission electron microscopy (TEM, JEOL JEM-2010). The TEM samples were prepared with a focused ion beam (FIB, FEI, Nova Nanolab). Te binding energies were determined using X-ray photoelectron spectroscopy (XPS; Kratos AXIS-NOVA) with Monochromatic Al-Ka (1486.6 eV) photon source and ultraviolet photoelectron spectroscopy (UPS, Kratos AXIS-NOVA) with a He-I photon source (21.2 eV). The C 1s peak at 286.5 eV was used for calibration purposes. Samples were sputtered with Ar gas to remove contamination and the native oxide on the thin film prior to analysis. Films were detached from the substrate using Torr Seal epoxy (Struers, ProntoFix) to examine their electronic transport properties; therefore, the rear surface of the film was examined. The room-temperature electrical conductivity (*σ*), Hall mobility (*µ*_Hall_), and carrier concentration (*n*_c_) of each as-deposited and annealed thin film were determined using a Hall-effect measurement unit (ECOPIA, HMS-5300) in the van der Pauw configuration. Seebeck coefficients (*S*) were determined from plots of measured Seebeck voltages as functions of temperature difference (<3 °C) across the specimen (*S* = ∆V/∆T) at 27°C using an a self-made S measurement system.

### Mechanical Exfoliation of Cu-Te Thin Film

**FIGURE 1:**
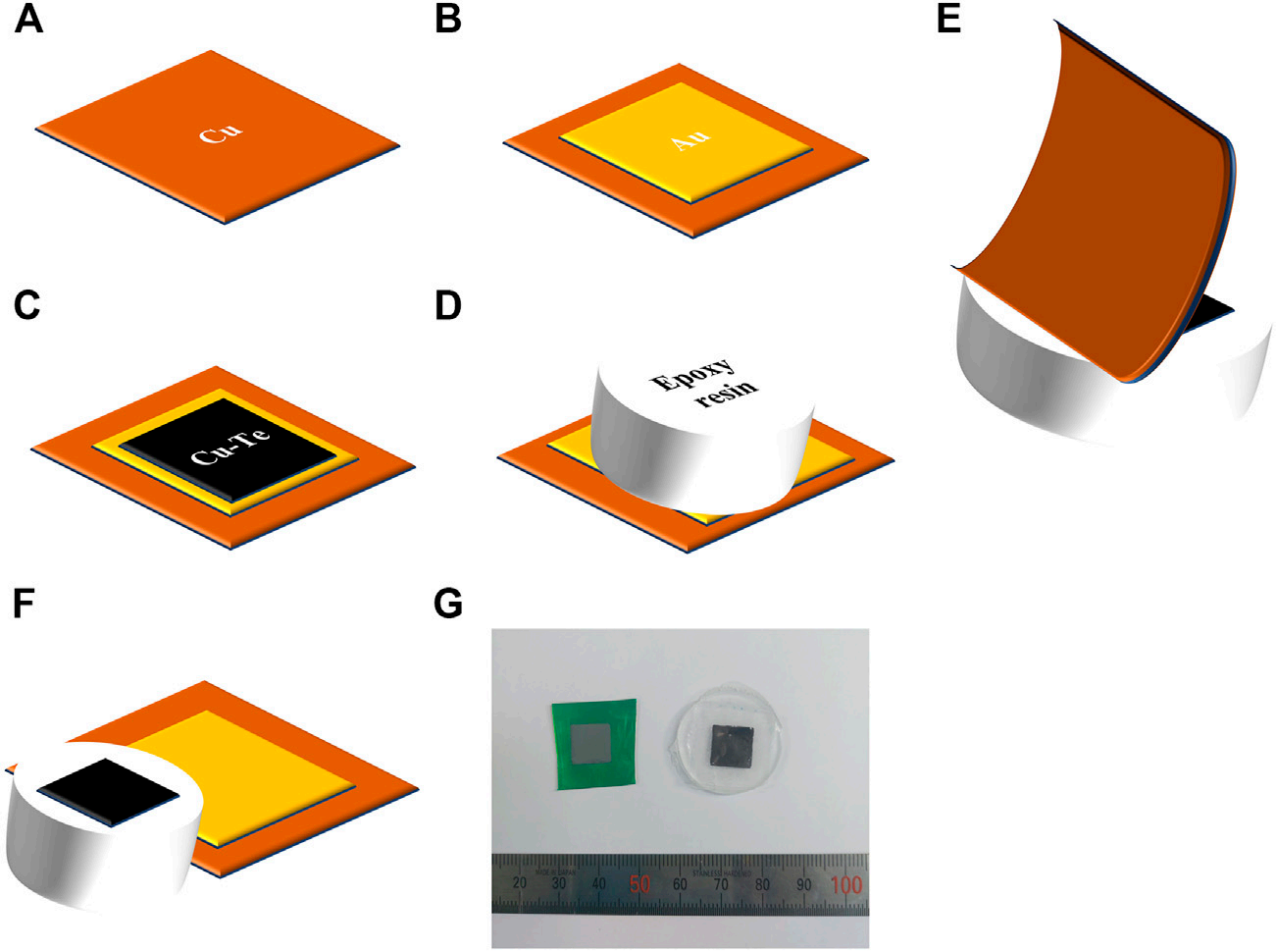
Schematic depicting mechanical exfoliation: **(A)** Cu foil, **(B)** Au seed layer obtained by e-beam evaporation, **(C)** Cu-Te electrodeposition, **(D)** pasting and drying of epoxy resin, **(E)** mechanical exfoliation, **(F)** after mechanical exfoliation, **(G)** photograph of electrodeposited Cu-Te thin film (left) and mechanically exfoliated film on epoxy resin (right).

The electrodeposited copper telluride thin films were mechanically exfoliated from the conducting substrate for investigating the electrical and thermoelectric properties Figures 1A–F is a scheme of the exfoliation process. Cu foil was purchased from ILJIN MATERIALS. Each Cu foil was sliced into pieces of dimensions 15 × 15 mm^2^ (Figure 1A). A Ti adhesive layer (20 nm) and Au seed layer (200 nm) were deposited using the E-beam evaporator after cleaning the Cu foil in 10%vol. H_2_SO_4_ to remove the native oxide layer of the Cu film (Figure 1B). Following the preparation of the substrate, Cu-Te thin films were deposited on it (Figure 1C). After rinsing and drying the sample, epoxy resin was pasted on the electrodeposited Cu-Te thin film, followed by hardening in air for 24 h (Figure 1D). Finally, the flexible Cu/Ti/Au foil substrate was exfoliated from the hardened epoxy resin on which the electrodeposited thin film was transferred (Figures 1E,F). This method enabled the exfoliation of the electrodeposited thin film from the conducting substrate without cracks on the surface, thereby allowing measurements of the electrical properties of the electrodeposited thin films. Figure 1G shows a photograph of the electrodeposited copper telluride thin film (left) and the transferred thin film on epoxy resin (right).

## Results and Discussion

### Copper Telluride Electrodeposition

**FIGURE 2:**
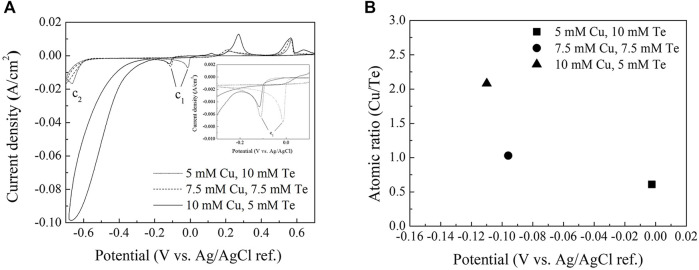
**(A)** Cyclic voltammograms of a Au/Ti/Si substrate in Cu(NO_3_)_2_·3H_2_O/TeO_2_/HNO_3_ between −0.7 and 0.7 V. The inset shows the same cyclic voltammograms between −0.3 and 0.05 V **(B)** EDS data for thin films deposited in each bath at various potentials (−2.5, −96, and −110 mV).

Figure 2A shows cyclic voltammograms of the copper telluride baths used in the electrodeposition experiments. The potential was swept in the cathodic direction from the open-circuit potential (OCP) at a scan rate of 10 mV/s between −700 and 700 mV (vs. Ag/AgCl sat.) in steps of 10 mV/s and then back to the OCP. CV revealed that the copper telluride (C1) reduction peak at around −0.1 V varies with concentration. Copper has a more noble reduction potential (E_0_ = 117 mV vs. Ag/AgCl sat.) than that of the Te ion (E_0_ = − 200 mV vs. Ag/AgCl sat.) (Rudnik and Kozłowski, 2013). However, the reduction peak shifted in the positive direction with decreasing Cu/Te bath salt ratio, which suggests that the Te underpotential deposition (UPD) reaction occurs on the Au substrate surface, as previously observed by Sorenson et al. (2001), which accelerates copper telluride deposition at a more noble potential. This result also suggests that any increase or decrease in the limiting current of the reduction peak (C1) during CV is mainly due to the concentration of Te ions near the working electrode surface.

Secondary peaks (C2) began to appear at approximately –600 mV when the potential was moved in the negative direction (vs. Ag/AgCl sat.), which corresponds to the formation of H_2_Te as given by (Kim et al., 2016)

$$Te+2H^{+}+2e^{-}\to H_{2}Te$$ (1)

Copper reduction appears to dominate after the copper telluride reduction peak (C1) in the 10 mM Cu + 5 mM Te bath, and the reduction peak corresponding to the formation of H_2_Te (C2) is no longer clearly observed.

Figure 2B displays the atomic ratios of copper to tellurium of deposits formed in each bath as determined by EDS. Cu_2_Te and CuTe stoichiometries were observed for thin films deposited in baths containing 10 mM Cu + 5 mM Te and 7.5 mM Cu + 7.5 mM Te, respectively. In contrast, the thin films deposited in the bath containing 5 mM Cu + 10 mM Te exhibited a 1:2 compositional ratio, which does not fit the stoichiometry of any intermetallic copper telluride compound; metallic Te and an intermetallic copper telluride compound possibly coexist based on this compositional ratio.

**FIGURE 3:**
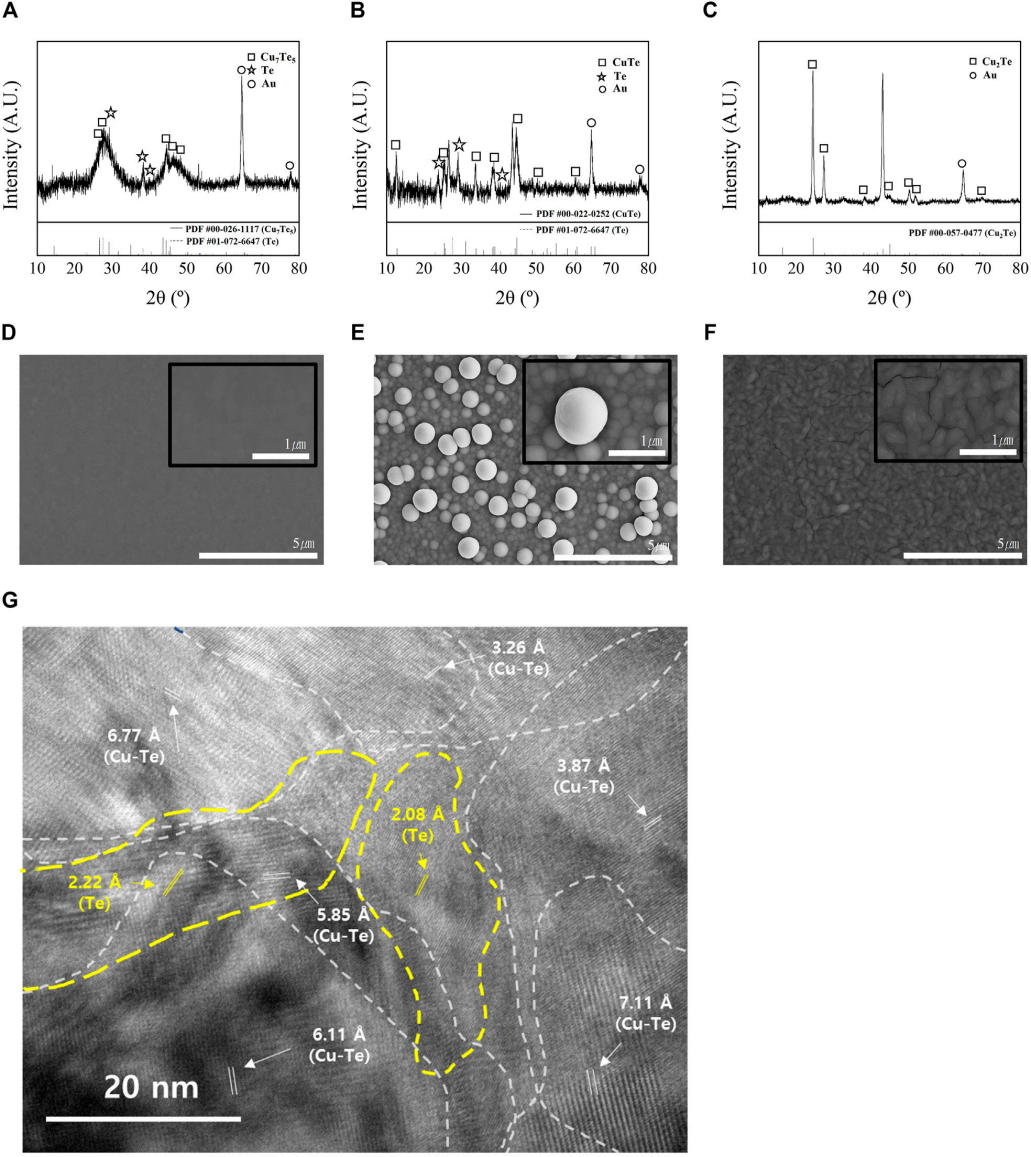
XRD patterns thin films formed at Cu/Te compositional ratios of **(A)** 0.5 (PDF #00-026-1117: Cu_7_Te_5_, PDF #01-072-6647: Te), **(B)** 1 (PDF #00-022-0252: CuTe), **(C)** 2 (PDF #00-057-0477: Cu_2_Te), and SEM images [10000x, 50000x (insets)] of thin films (1 mm) at Cu/Te compositional ratios of **(D)** 0.5 (Cu_7_Te_5_+Te), **(E)** 1 (CuTe), **(F)** 2 (Cu_2_Te). **(G)** HR-TEM (250kx) image of thin films formed at a Cu/Te compositional ratio of 0.5.

Figure 3 shows XRD patterns and SEM images of the electrodeposits formed in each bath. The reference powder diffraction files (PDFs) for copper tellurides and Te are: #00-026-1117 (Cu_7_Te_5_), #01-072-6647 (Te), #00-022-0252 (CuTe), and #00-057-0477 (Cu_2_Te). The XRD pattern of the thin film with Cu/Te = 0.5 is shown in Figure 3A, which reveals the presence of both Cu_7_Te_5_ and Te, and the broad diffuse peaks in the XRD spectrum suggest that the film has a nanocrystalline phase. The HR-TEM image of the thin film with Cu/Te = 0.5 (Figure 3G) is consistent with the XRD pattern of the sample. The HR-TEM image at 250kx magnification does not show clear grain boundaries, but shows various orientations of 100 nm-sized grains with different d spacings that overlap each other. Figure 3D shows the SEM image of the Cu_0.5_Te thin film, which exhibits a smooth and dense surface morphology, while Figure 3B shows the XRD pattern of the thin film with Cu/Te = 1, which reveals the presence of crystalline intermetallic CuTe, and small amounts of Te. Unlike the surface morphology of the Cu_0.5_Te thin film, which exhibits a smooth and dense surface, the surface morphology of the CuTe thin film (Figure 2E) can be described as a cluster of small nanoparticles forming the thin film with spherical micrometer-sized particles on the top, akin to the formation of other chalcogenides under low-pH conditions (Saha et al., 2020). The thin film with Cu/Te = 2 (Figure 3C) shows obvious crystallinity that corresponds to intermetallic Cu_2_Te; this thin film exhibits an ellipsoidal particle morphology with cracks that may result from internal tensile stresses (Figure 3F) (Eliaz et al., 2005). Materials with high carrier concentrations are generally less resistive than those with low carrier concentrations; hence, the observed cracks may adversely affect thin-film resistivity, resulting in poor thermoelectric properties.

**FIGURE 4:**
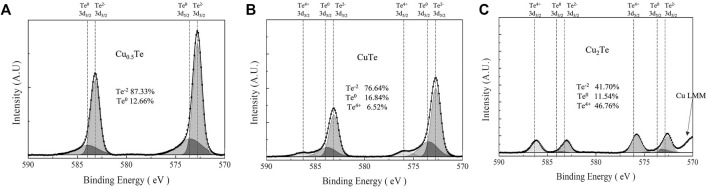
Te 3d XPS spectra acquired from thin films at each Cu/Te compositional ratios of **(A)** 0.5, **(B)** 1, **(C)** 2.

The Te 3d XPS spectra of the thin films (Figure 4) show Te binding energies that correspond to Te^4+^ (576.1 eV, 3d_5/2_), Te^0^ (573.0 eV, 3d_5/2_), Te^2−^ (572.6 eV, 3d_5/2_), consistent with TeO_2_, Te metal, and Te bound to Cu, respectively. The Te 3d XPS spectrum of the Cu_0.5_Te thin film (Figure 4A) shows high intensity Te^2−^ 3d peaks (87.3%) and less intense Te^0^ 3d peaks (12.7%) that corresponds to the left shoulders of the major peaks, with Te^4+^ 3d peaks noticeably absent. These data confirm that intermetallic Cu_7_Te_5_ and a small amount of Te metal coexist without TeO_2_ in the thin film. The XPS pattern of the CuTe thin film is shown in Figure 4B, in which high intensity Te^2−^ 3d peaks (76.64%) and smaller Te^0^ 3d (16.84%) and Te^4+^ 3d (6.52%) peaks are observed, which implies that the CuTe thin film contains intermetallic CuTe, Te metal, and a small amount of TeO_2_. Compared to the Cu_0.5_Te thin film, the CuTe thin film exhibits a somewhat larger Te^0^ peak, with a small amount of TeO_2_ also observed. The Te 3d XPS spectrum of the Cu_2_Te thin film is shown in Figure 4C. This film exhibits the largest Te^4+^ 3d peaks (46.76%) and the smallest Te^2−^ 3d (41.70%) and Te^0^ 3d (11.54%) peaks among the three thin film samples; it also shows a Cu LMM peak near 570 eV, consistent with the presence of Cu metal.

### Electrical and Thermoelectrical Properties of Electrodeposited Copper Telluride Thin Films

**FIGURE 5:**
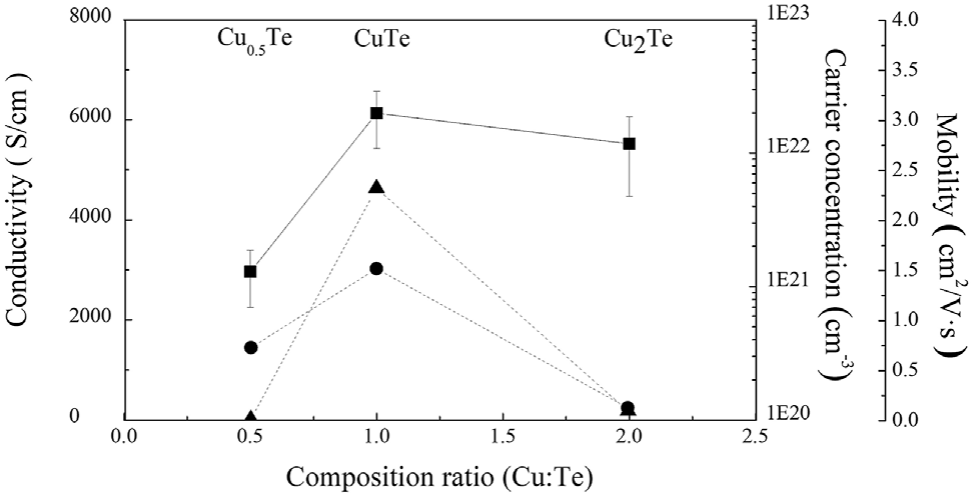
Electrical properties of thin films deposited by electrodeposition as functions of the Cu/Te compositional ratio [▲ = Conductivity (S/cm), ■: Carrier concentration (cm^−3^), ● = mobility (cm^2^/V s)].

Hall-effect measurements using the van der Pauw method were used to examine the electrical properties of the electrodeposited thin films after exfoliation. All films were deposited with the same applied charge at 5C to produce films with the same 2.5-μm thickness. However, we were unable to form a 2.5-μm-thick Cu_2_Te film without delaminating it from the substrate prior to exfoliation due to internal stress. Consequently, a 1-μm-thick Cu_2_Te sample was exfoliated from the substrate in this case. Figure 5 displays the electrical characteristics of the copper telluride thin films, including carrier concentration, conductivity, and carrier mobility. All electrodeposited copper telluride thin films exhibited relatively high carrier concentrations (10^21^–10^23^ cm^−3^) as p-type semiconductors, which is consistent with the carrier concentrations of other copper chalcogenides and is ascribable to copper vacancies (Dorfs et al., 2011; Luther et al., 2011; Kriegel et al., 2012). The Cu_0.5_Te thin film is the least conductive (20 S cm^−1^), which is due to electron scattering induced by the interface between the two phases and the large proportion of grain boundaries. The CuTe thin film deposited in this work exhibits the highest electrical conductivity (4,632 S cm^−1^) among previously reported CuTe thin films. Bhuvaneswari et al. (2017) reported electrical conductivities of 384–714 S cm^−1^ for 1-μm-thick CuTe thin films deposited by vacuum evaporation. The conductivity of the CuTe thin film prepared in the current work is similar to that reported previously for bulk copper telluride (4200 S cm^−1^) (He et al., 2015). Cu_2_Te thin films are poorly conductive and have low carrier mobilities compared to their carrier concentrations.As discussed above (*vide supra*), the low conductivity and carrier mobility is possibly attributable to cracks induced by stresses inside the thin film, as observed in the corresponding SEM image (Figure 3F).

Table 1 lists the thermoelectric properties of the electrodeposited thin films and others reported in the literature (Mansour et al., 1986; Nethravathi et al., 2014; Ghosh et al., 2016; Mukherjee et al., 2019; Mukherjee et al., 2020), including Seebeck coefficients, conductivities, and calculated power factors. The electrodeposited thin films have somewhat larger Seebeck coefficient than their bulk counterparts. Previously reported copper telluride bulk and thin film samples (Mansour et al., 1986; Nethravathi et al., 2014; Ghosh et al., 2016; Mukherjee et al., 2019; Mukherjee et al., 2020) exhibited relatively low Seebeck coefficients of between 4 and 40 μV K^−1^. While electrodeposited Cu_0.5_Te showed a particularly high Seebeck coefficient (346 μV K^−1^), the Cu_0.5_Te thin film is extremely poorly electrically conductive, which results in a low power factor (2.48 μW cm^−1^ K^−1^). On the contrary, CuTe exhibited the highest power factor (7.71 μW cm^−1^ K^−1^) despite its low Seebeck coefficient, which is ascribable to its high conductivity. The CuTe thin film has a high power factor because its conductivity is of similar order to that of the bulk sample but its Seebeck coefficient is considerably higher. In contrast, the Cu_2_Te sample shows the lowest power factor owing to its low Seebeck coefficient and conductivity. While Seebeck coefficient and conductivity exist in a trade-off relationship, the Cu_2_Te sample has low values owing to its cracked surface.

**TABLE 1:** Thermoelectric properties of copper tellurides determined by Hall-effect measurement (this work) and the *S* measurement system at room temperature.

Compositional ratio (Cu/Te	Seebeck coefficient (μV K^−1^	Conductivity (S cm^−1^	Power factor (μW·cm^−1^K^−1^	Shape/Synthesis method	
0.5	346	20.74	2.48	Thin film/Electrodeposition	This work
1	40.8	4632	7.71	Thin film/Electrodeposition	This work
2	53.8	178.71	0.52	Thin film/Electrodeposition	This work
2	25	4,000	2.5	Bulk/Hot pressing	Nethravathi et al. (2014)
2	15	8,000	1.8	Bulk/Solid state reaction	Mukherjee et al. (2020)
2	15	6,427	1.6	Bulk/Solid state reaction	Mansour et al. (1986)
2	4	1,000	0.0016	Bulk/Melting	Ghosh et al. (2016)
1.75	40	2,550	4.1	Thin film/Electrodeposition	Mukherjee et al. (2019)

**FIGURE 6:**
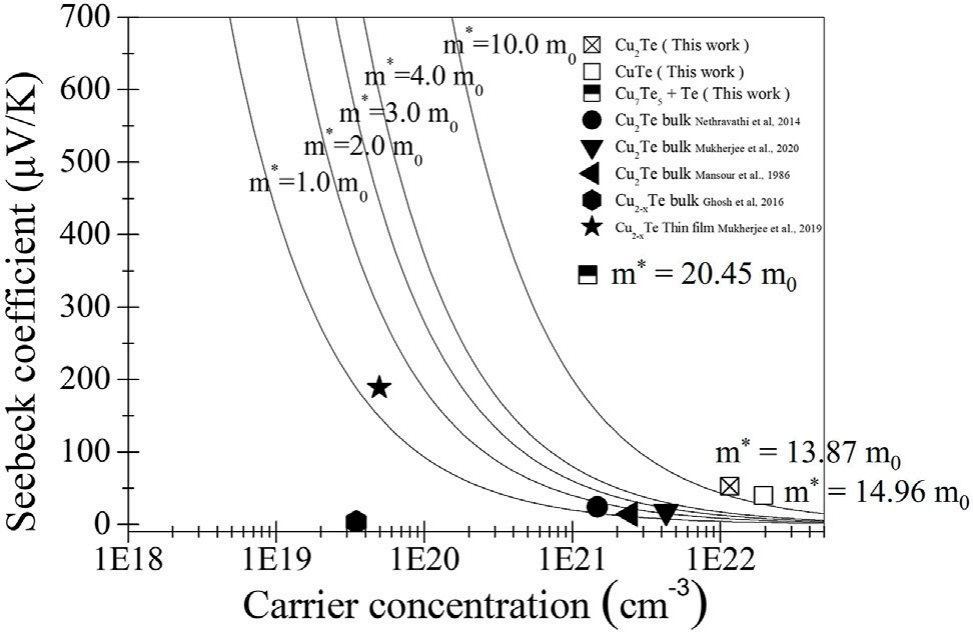
Seebeck coefficients of copper telluride thin films deposited by electrodeposition and other methods as functions of carrier concentration (Pisarenko plots). Square symbols correspond to thin films prepared in this work. The solid lines are curves calculated for m^*^ = 1, 2, 3, 4, and 10 m_0_.

Pisarenko plots, which show relationships between Seebeck coefficients and carrier concentrations determined through Hall-effect measurements, are shown in Figure 6. The carrier effective masses of the electrodeposited thin films were calculated assuming a simple band structure using the following electron transport relationship:

$$S=\frac{8\pi^{2}k_{b}^{2}}{3eh^{2}}{\left(\frac{\pi }{3n}\right)}^{2/3}m^{*}T$$ (2)

where S is the Seebeck coefficient, *k*_*b*_ is Boltzmann’s constant, *h* is Planck’s constant, n is carrier concentration, m^*^ is the carrier effective mass, and T is temperature.

The solid curves shown in Figure 6 correspond to m^*^ = 1.0, 2.0, 3.0, 4.0, and 10.0 m_0_, and are provided for guidance. The carrier concentrations and Seebeck coefficients of literature samples provided in Table 1 are also plotted in the figure for comparison. All electrodeposited thin films show significantly high carrier effective masses compared to literature values, which is mainly due to the formation of potential wells at the interfaces between the two different phases, namely intermetallic copper telluride and tellurium metal. The potential wells of the two phases are responsible for the high Seebeck coefficients compared to those reported in the literature, despite their high carrier concentrations. This effect provides high power factors because high Seebeck coefficients are not traded off against thin film conductivity.

**FIGURE 7:**
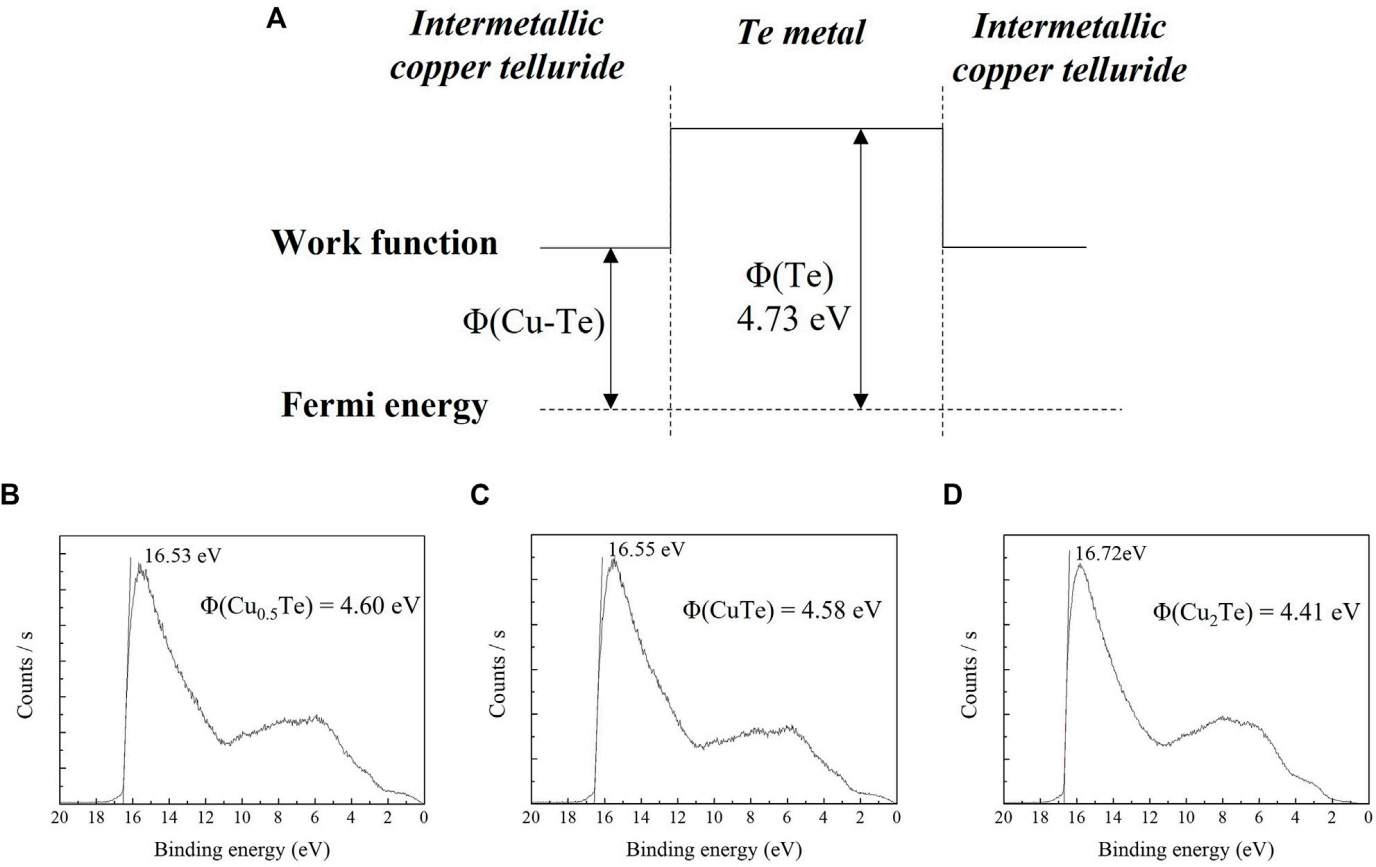
**(A)** Potential well scheme at the interface between the intermetallic copper telluride and Te metal phases. UPS spectra acquired for calculating the work functions of: **(B)** Cu_0.5_Te, **(C)** CuTe, and **(D)** Cu_2_Te.

Figure 7A shows the potential well scheme resulting from the difference in the work function of intermetallic CuTe and that of Te metal at the interface between the two phases. As the two phases have different work functions, the potential difference creates a potential well that affects the thermoelectric properties of the thin film due to carrier filtering. There is a close relationship between the high thermoelectric performance and carrier effective mass. The carrier effective mass is proportional to the Seebeck coefficient. However, typically, a higher carrier effective mass implies a lower conductivity of the material. To achieve high-performance thermoelectrics, increasing the carrier effective mass without degrading the conductivity of the material is essential. The carrier effective mass is expressed as m^*^ = N_v_^2/3^m_b_^*^, where N_v_ includes the orbital degeneracy and m_b_^*^ is the average carrier effective mass for single-band valley degeneration. Thus, the carrier effective mass can be increased by introducing a band offset in the material without lowering the conductivity of the material (Pei et al., 2011). The potential difference between the Te metal and intermetallic CuTe was determined by UPS. Figures 7B–D show UPS spectra for intermetallic Cu_0.5_Te, CuTe, and Cu_2_Te, respectively, with the work functions of the various copper tellurides calculated using the equation:

$$\Phi =h\nu -E_{cutoff}$$ (3)

where Φ is the work function of the material, *ν* is the frequency of the He beam, and *E*_*cutoff*_ is the secondary electron cut-off energy of the material. The work functions of the copper tellurides were determined to be 4.60 eV (Cu_0.5_Te), 4.58 eV (CuTe), and 4.41 eV (Cu_2_Te). As the work function of Te metal is 4.73 eV (Michaelson, 1950), the potential differences between the two phases are 0.13, 0.15, and 0.32 eV, respectively. The band offset between the copper telluride and Te metal increases the Seebeck coefficient without sacrificing the conductivity of the thin film owing to carrier filtering. The CuTe thin film sample exhibits a particularly high-power factor due to Te precipitation inside the thin film, which results carrier-energy filtering.

## Conclusion

In this study, stoichiometric intermetallic copper telluride thin films were successfully electrodeposited in aqueous solutions prepared using Cu(NO_3_)_2_·3H_2_O, TeO_2_, and HNO_3_. CV was used to determine appropriate electrodeposition potentials in a three-electrode system, while the thin film deposited in each bath was analyzed by SEM, EDS, and XRD. The electrodeposited thin films were exfoliated from the substrate and mounted on epoxy resin, and their electrical and thermoelectrical properties were examined using Hall-effect and thermoelectric measurements. Thin films deposited in baths at different potentials showed compositions and structures that affect their electrical and thermoelectric properties. The thin film with Cu/Te = 2 contains crystalline intermetallic Cu_2_Te, while that with Cu/Te = 1 contains crystalline intermetallic CuTe and Te. In contrast, the thin film with Cu/Te = 0.5 exhibited a broad XRD pattern that corresponds to intermetallic Cu_7_Te_5_ and Te metal, suggestive of a nanocrystalline structure and the coexistence of Te metal. XPS also revealed the coexistence of Te metal in the film, with Te^0^ peaks observed for thin films with Cu/Te ratios of 1 and 0.5. In other words, the coexistence of these two phases creates a band offset that improves thermoelectric properties by increasing the Seebeck coefficient of the thin film without sacrificing its conductivity. The band offset resulted in CuTe exhibiting the highest power factor of 7.71 μW cm^−1^ K^−1^, with the potential difference between intermetallic CuTe and Te metal determined to be 0.15 eV by UPS.

## Data Availability

The original contributions presented in the study are included in the article/supplementary material, further inquiries can be directed to the corresponding authors.
